# Physiotherapy management of children with cerebral palsy in low- and middle-income countries: a scoping review protocol

**DOI:** 10.1186/s13643-023-02280-8

**Published:** 2023-07-01

**Authors:** Noxolo E. Duma, Mbuzeleni Hlongwa, Natalie Benjamin-Damons, Khumbulani W. Hlongwana

**Affiliations:** 1 Discipline of Public Health Medicine, School of Nursing and Public Health, University of KwaZulu-Natal, Durban, South Africa; 2 Public Health, Societies and Belonging, Human Sciences Research Council, Pretoria, South Africa; 3 Department of Physiotherapy, Faculty of Health Sciences, University of the Witwatersrand, Johannesburg, South Africa; 4 Cancer & Infectious Diseases Epidemiology Research Unit (CIDERU), College of Health Sciences, University of KwaZulu-Natal, Durban, South Africa

**Keywords:** Cerebral palsy, Physiotherapy, Physical therapy, Management, Intervention, LMICs

## Abstract

**Introduction:**

Cerebral Palsy (CP) is the most common childhood physical disability worldwide. Approximately 1.5 to 4 children per live births live with CP, globally. There have been no specific treatments that can reverse the brain damage responsible for the complex clinical dysfunctions of CP. There are, however, several interventions that are currently being used by physiotherapists, most of which are deemed to be ineffective and unnecessary. We will conduct a scoping review aimed at mapping evidence on the physiotherapy management of children living with CP in low- and middle-income countries (LMICs).

**Methods:**

The scoping review will be guided by the Arksey and O’Malley and Levac et al. frameworks. The databases that will be used to search for literature include PubMed, MEDLINE, CINAHL, EBSCOhost, Web of Science, and ProQuest One Academic and Scopus. Gray literature articles will also be included in this review, provided they meet our inclusion criteria. The Preferred Reporting Items for Systematic Reviews and Meta-Analysis: Extension for Scoping Reviews (PRIMSA-ScR) guideline will be used to report the results of the scoping review. The screened results will be reported using the PRISMA flow diagram guidelines, and the results will be charted using an electronic data charting form and analyzed using thematic analysis.

**Discussion:**

Understanding how physiotherapists manage children with CP in LMICs is essential for the development of internationally sound, yet locally relevant, intervention strategy for physiotherapists. It is anticipated that the results of the scoping review will inform the thinking geared towards the development of a contextualised evidence-based framework for physiotherapists to effectively manage CP in children.

**Systematic review registration:**

Open Science Framework. https://doi.org/10.17605/OSF.IO/VTJ84

## Background

Cerebral palsy (CP) is the most common childhood physical disability worldwide [1], with approximately 1.5 to 4 children per 1000 live births living with CP, globally [2]. CP is a group of permanent disorders of the development of movement and posture, causing activity limitations that are attributed to non-progressive disturbances that occurred in the developing foetal or infant brain [3]. There have been no specific treatments that can reverse the brain damage responsible for the complex clinical dysfunctions of CP [4]. There are, however, a number of interventions such as neurorehabilitation, orthopedic surgery, and medication, which are aimed at remediating musculoskeletal changes caused by the injury and improves the activity level, participation and therefore, the quality of life [4]. However, the number of interventions currently being implemented by physiotherapists are deemed ineffective and unnecessary [5]. In 2017, the World Health Organization (WHO) launched the rehabilitation 2030 initiative, which calls for all stakeholders, worldwide, to collaboratively improve research on rehabilitation [6].

In the USA, the prevalence of CP is reported at 3 per 1000 live births [7]. In LMICs such as India and Brazil, the prevalence was reported to be 4.37 and 5 per 1000, respectively [8, 9]. In a Ugandan study, a prevalence of 2.9 per 1000 was reported [10]. However, a higher prevalence of 10 per 1000 was reported in a study conducted in South Africa (SA) [11].

The proportion of severe cases of CP is very high in LMICs, and children in LMICs lack adequate access to rehabilitation [12]. Little is reported on CP in the African context, thus basic care is lacking due to the paucity of available interventions and relevant guidelines [13]. Furtado et al. [14] conducted a scoping review on the physiotherapy management of CP which focused on reducing impairments and activity limitations; however, participation and environmental factors, which form part of the International Classification of Function and Disability, and Health (ICF) Framework, were not included in the review. A clinical practice guideline (CPG) was developed in Australia, where the stakeholders conceded that methods used to manage children with CP were outdated and possibly harmful [15]. CP is one of the disabilities that add to the diseases burden and further impose strain on the health sector [16]. It is against this backdrop that concerted efforts should be directed at developing evidence-based interventions to mitigate the burden of CP in children.

Physiotherapy plays a critical role in the management of CP, and almost all persons diagnosed with CP are referred for physiotherapy services [17]. Considering the role played by physiotherapists in the management of CP, it is of utmost importance that physiotherapists base their therapeutic interventions on the most recent internationally benchmarked, yet locally relevant evidence [5]. This scoping review protocol proposes to map evidence on the physiotherapy management of children living with CP in LMICs. It is anticipated that the results will provide evidence to guide future research and reveal gaps to be addressed to improve the management of children living with CP. This review will enable the implementation of guidelines to manage CP and will also add to the body of evidence in the field of CP.

## Methods

### Study design

We will conduct a scoping review of peer-reviewed published studies and gray literature articles (which is a range of documents not controlled by commercial publishing organisations namely; theses, policy literature, government documents, and reports [18]) to map evidence on the physiotherapy management of children living with CP in LMICs. We will use Arskey and O’Malley framework [19] and Levac et al. [20] to guide our scoping review. The following are the steps that we will follow in conducting our scoping review; (a) to identify the research question, (b) identifying relevant studies, (c) to select studies, (d) to chart the data, and (e) to collate, summarize, and report data [19].

The Preferred Reporting Items for Systematic Reviews and Meta-Analysis: Extension for Scoping Reviews (PRIMSA-ScR) [21] guideline will be used to report the results of the scoping review.

### Identifying the research question

#### Research question

What is the current evidence on the physiotherapy management of children with cerebral palsy in LMICs? To determine the suitability of the research question, the Population, Concept, and Context (PCC) framework will be used (Table 1).

**Table 1:** PCC for determining the eligibility of the research question

Criteria	Determinants
Population	Children with CP
Concept	Physiotherapy management
Context	LMICs

#### Identifying relevant studies

An advanced search through electronic databases such as PubMed, MEDLINE, CINAHL, EBSCOhost, Web of Science, ProQuest One Academic, and Scopus will be conducted. A search strategy will be developed by the Principal Investigator (PI), in consultation with the librarian to ensure the quality of our search strategy and that correct medical subject headings (MeSH) terms are used. The keywords that will be used to search for relevant articles through database search will include: “cerebral palsy,” “physiotherapy management,” “physical therapy management, physiotherapy intervention, and physical therapy intervention.” All studies suitable for inclusion will have their reference lists evaluated for potential further research. An initial search of PubMed will be undertaken, followed by an analysis of title and abstract text words, as well as index terms used to characterize the publications. This will guide the creation of a search strategy that is specific to each information source. We have piloted a PubMed database search to understand the feasibility of this review (Table 2).

**Table 2:** Pilot database search results

Criteria	Search Date	Search Engine	No. Retrieved
((("physical therapy modalities"[MeSH Terms] OR ("physical"[All Fields] AND "therapy"[All Fields] AND "modalities"[All Fields]) OR "physical therapy modalities"[All Fields] OR "physiotherapies"[All Fields] OR "physiotherapy"[All Fields] OR ("physical therapy modalities"[MeSH Terms] OR ("physical"[All Fields] AND "therapy"[All Fields] AND "modalities"[All Fields]) OR "physical therapy modalities"[All Fields] OR ("physical"[All Fields] AND "therapy"[All Fields]) OR "physical therapy"[All Fields])) AND ("manage"[All Fields] OR "managed"[All Fields] OR "management s"[All Fields] OR "managements"[All Fields] OR "manager"[All Fields] OR "manager s"[All Fields] OR "managers"[All Fields] OR "manages"[All Fields] OR "managing"[All Fields] OR "management"[All Fields] OR "organization and administration"[MeSH Terms] OR ("organization"[All Fields] AND "administration"[All Fields]) OR "organization and administration"[All Fields] OR "management"[All Fields] OR "disease management"[MeSH Terms] OR ("disease"[All Fields] AND "management"[All Fields]) OR "disease management"[All Fields])) OR ("intervention s"[All Fields] OR "interventions"[All Fields] OR "interventive"[All Fields] OR "methods"[MeSH Terms] OR "methods"[All Fields] OR "intervention"[All Fields] OR "interventional"[All Fields])) AND ("cerebral palsy"[MeSH Terms] OR ("cerebral"[All Fields] AND "palsy"[All Fields]) OR "cerebral palsy"[All Fields])	27/05/2022	PubMed	14 801

#### Inclusion criteria

- ⚬ Articles that are published in English.
- ⚬ Articles presenting evidence on physiotherapy management on children living with CP.
- ⚬ Articles that are conducted in LMICs.
- ⚬ Articles published between 2012 and 2022.

#### Exclusion criteria

- ⚬ Articles from scoping reviews, meta-analysis, and rapid reviews.
- ⚬ Articles conducted outside LMICs.

#### Study selection

**Fig. 1:**
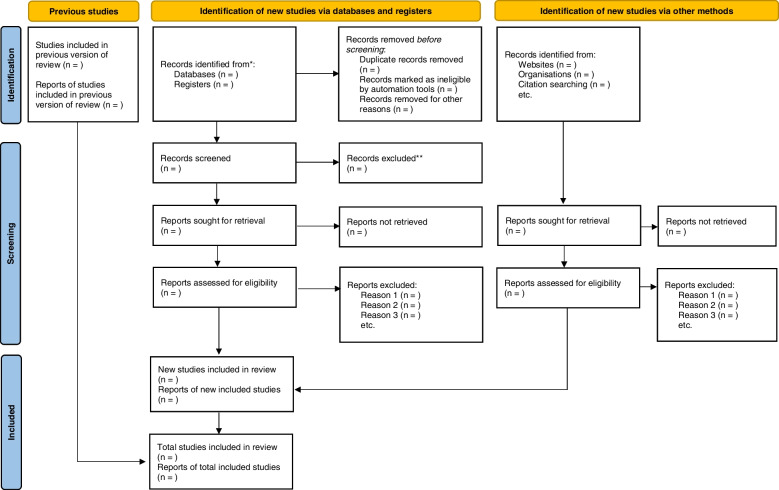
The PRISMA 2020 flow diagram for reporting screening results [22]

The articles will be screened in three stages, i.e., title, abstract, and full-article screening. The PI will screen all the titles that are retrieved from the database searching. Titles found to be eligible for inclusion will be exported to EndNote version 20 library. The Endnote library will be used to identify any duplicates for deletion. The EndNote library’s “find full-text” option will be used to automatically obtain PDFs of studies. In cases where PDFs could not be retrieved, a librarian will be contacted to provide support. Additionally, the authors of the said publications will be contacted and requested to share the full-texts PDFs. Two independent reviewers will conduct the abstract and full-article screenings, in line with the inclusion and exclusion criteria. After screening has been completed at each stage (abstract/full article), the two reviewers will then meet and discuss the screening outcomes, including any discrepancies that may arise. Any differences between the two independent reviewers will be handled through conversation or collaboration with a third reviewer. The PRISMA flow diagram will be used for reporting the screening results at each step of the screening process (Fig. 1).

#### Charting the data

An electronic data charting form (Google forms) will be used to extract the information, using the narrative review of the included articles. The key information to be extracted will include the following: (a) author(s) and date of publication, (b) aim(s) or research questions, (c) primary source data, (d) study population, (e) mean age of participants, (f) gender, (g) percentage of women, (h) percentage of men, (i) geographic setting (rural/urban), (j) study design, (k) type of Intervention and outcomes, (l) most relevant finding, (m) most significant finding, and (n) study limitations and implications, as well as (o) interpretations and conclusions from the authors. The reviewers will conduct a pilot of the charting form to ensure that all sections are covered. Modification of the form will be conducted which will be based on the reviewers’ comments.

#### Collating, summarizing, and reporting the results

We will present a narrative account of the findings from the eligible studies, presenting the main concepts from the included articles in line with the research question. The reviewers will consider the use of google forms during data extraction to assist with organizing data. A thematic analysis will be carried out collectively by reviewers to extract relevant outcomes using NVIVO version 12 for theme extraction of the included articles. The themes will be structured around these outcomes: interventions, sample size, participants, research methods, outcomes, and evidence relating to effectiveness [19]. Reviewers will read and familiarize themselves with the data of all eligible studies, considering the research question, aim, and the anticipated results. Coding of the findings of the included studies line-by-line will be conducted. After codes have been extracted, themes and patterns will be identified, and these codes will be categorized into major themes. The researcher will relook at the themes and identify gaps which may warrant future research. The inferences of the study results for future research, policy, and practices, will be examined and reported on.

#### Quality appraisal

The Mixed Methods Appraisal Tool (MMAT) version 2018 will be used to evaluate the quality of the eligible studies [23]. Two independent reviewers (PI and co-screener) will carry out the quality appraisal process. A quality appraisal will be carried out to examine the strengths, weaknesses, potential bias of studies, and quality of research evidence presented for each article. This study will assess all the categories which are (a) qualitative studies, (b) quantitative randomized controlled trials, (c) quantitative non-randomized trials, (d) quantitative descriptive studies, and (e) mixed methods studies [23].

The MMAT tool will be used to examine the relevance of the aim of the study, methodology, study design, data collection, data analysis, presentation of findings, authors’ discussions and conclusion, and the overall quality of the study.

The gray literature will be appraised using the Authority, Accuracy, Coverage, Objectivity, Date, Significance (AACODS) checklist, which was designed to enable evaluation and critical appraisal of gray literature [24]. The checklist uses a series of questions under each of the headings and requires the user to assess aspects like whether the author is from a reputable organization, whether the aims and methodology are clearly stated, and whether the date is included [24].

The percentage for grading the quality of the evidence is as follows: (a) ≤ 50% will depict low quality, (b) 51–75% will depict average quality, and (c) 76–100% will depict high quality evidence. Should a study score low during the quality appraisal process, it will still be included in the review as the MMAT tool discourages the exclusion of the studies with low methodological quality [23]. This quality appraisal will enable the researchers to appraise a variety of study methods namely; qualitative, randomized trials, non-randomized trials, quantitative, and mixed methods [23].

#### Ethical considerations and dissemination

No ethical approval is needed for this study as it will not include animals or human participants. The findings of the scoping review will be peer-reviewed and disseminated through publication in an accredited journal, in print and through peer presentations, conferences, and congresses.

## Discussion

Physiotherapy plays a major role in the management of children living with CP. In LMICs, the prevalence of CP is high, it is reported at 3.1 per 1000 [25–28], and children with CP in these countries present with more severe limitations [29–31]. The high prevalence of CP warrants effective evidence-based physiotherapy strategies to manage and prevent further disabilities in children living with CP. Most children living with CP may not receive the therapy they need due to inadequate staffing and high patient load [17]. Few evidence-based interventions have been evaluated in LMICs [32]. Considering the importance of the role played by physiotherapists in the management of CP, it is of utmost importance that the management is based on current locally relevant evidence.

The proposed scoping review will map evidence on the physiotherapy management of children living with cerebral palsy. The evidence that will be sourced during the scoping review will help guide the development of an evidence-based management framework. This scoping review is part of a larger study that seeks to propose a framework geared towards addressing the needs of children living with CP. This scoping review will synthesize the existing evidence and reveal gaps in research, with a view to help guide the methodology of the main study.

Finding relevant studies that will map out the evidence on the physiotherapy management of children living with CP is anticipated. The findings of the study are anticipated to assist policymakers, advocacy groups, and task-teams to develop a practice guideline, which will standardize the physiotherapy management, in order to assist in preventing further disabilities in children living with CP.

### Strengths and limitations of this study

The strengths of this protocol include the following:

- ➢ The scoping review will be conducted using an established methodology
- ➢ An advanced search strategy used will include gray literature.
- ➢ The screened and included articles will go through the quality appraisal process using the MMAT tool to assess the quality of the eligible articles, in order to ensure trustworthiness, its value and relevance.
- ➢ However, the fact that only studies published in English will be included in this scoping review is an important limitation, hence studies published in any other language will be missed.

## Data Availability

The data that will be analyzed will be included in the scoping review article that will be published.

## Abbreviations

AACODS: Authority, Accuracy, Coverage, Objectivity, Date, Significance
CP: Cerebral palsy
LMICs: Low- and middle-income countries
MMAT: Mixed Methods Appraisal Tool
PRISMA: Preferred Reporting Items for Systematic Reviews And Meta-Analysis
WHO: World Health Organization
